# Clinical Significance of Reticulocyte Hemoglobin Content in the Diagnosis of Iron Deficiency Anemia

**DOI:** 10.4274/Tjh.2012.0107

**Published:** 2013-06-05

**Authors:** Mustafa Karagülle, Eren Gündüz, Fezan Şahin Mutlu, Meltem Olga Akay

**Affiliations:** 1 Eskişehir Osmangazi University Medical School, Department of Hematology, Eskişehir, Turkey; 2 Eskişehir Osmangazi University Medical School, Department of Biostatistics, Eskişehir, Turkey

**Keywords:** Iron deficiency anemia, Reticulocyte hemoglobin content, diagnosis

## Abstract

**Objective:** The aim of this study was to evaluate the clinical significance of reticulocyte hemoglobin content (CHr) in the diagnosis of iron deficiency anemia (IDA) and to compare it with other conventional iron parameters.

**Materials and Methods:** A total of 32 female patients with IDA (serum hemoglobin <120 g/L and serum ferritin <20 ng/ mL) and 18 female patients with iron deficiency (serum hemoglobin > 120 g/L and serum ferritin <20 ng/mL) were enrolled.

**Results:** CHr was 24.95±3.92 pg in female patients with IDA and 29.93±2.96 pg in female patients with iron deficiency. CHr showed a significant positive correlation with hemoglobin, mean corpuscular volume, mean corpuscular hemoglobin, mean corpuscular hemoglobin concentration, serum iron, and transferrin saturation and a significant negative correlation with transferrin and total iron-binding capacity. The cut-off value of CHr for detecting IDA was 29 pg.

**Conclusion:** Our data demonstrate that CHr is a useful parameter that can be confidently used in the diagnosis of IDA, and a CHr cut-off value of 29 pg predicts IDA.

**Conflict of interest:**None declared.

## Abstract (tr)

**Amaç:** Bu çalışmanın amacı, DEA tanısında retikülosit hemoglobin içeriğinin (CHr) klinik önemini belirlemek ve diğer konvansiyonel demir parametreleri ile karşılaştırmaktır.

**Gereç ve Yöntemler:** Çalışmaya demir eksikliği (serum hemoglobin >120 g/L ve serum ferritin <20 ng/mL) olan 18 kadın hasta ve demir eksikliği anemisi (serum hemoglobin <120 g/L ve serum ferritin <20 ng/mL) olan 32 kadın hasta dahil edildi.

**Bulgular:** Demir eksikliği anemisi olan hastalarda retikülosit hemoglobin içeriği 24,95±3,92 pg, demir eksikliği olan hastalarda ise 29,93±2,96 pg idi. CHr; hemoglobin, MCV, MCH, MCHC, serum demiri ve transferrin saturasyonu ile anlamlı pozitif korelasyon; transferrin ve total demir bağlama kapasitesi ile anlamlı negatif korelasyon gösterdi. DEA tanısı için CHr cut-off değeri 29 pg olarak saptandı.

**Sonuç:** Bizim verilerimiz, CHr’nin DEA tanısında güvenle kullanılabilecek faydalı bir parametre olduğunu ve 29 pg olan CHr cut-off değerinin demir eksikliği anemisini öngördüğünü göstermektedir.

## INTRODUCTION

Iron deficiency anemia (IDA) is the most common form of nutritional anemia worldwide [1]. Various biochemical parameters are used to diagnose IDA, including ferritin, transferrin saturation (TS), serum iron, and mean corpuscular volume (MCV). Despite the availability of these parameters, their validity for the diagnosis of IDA is still debatable. Serum ferritin, the most specific indicator of iron deficiency, is an acute phase reactant and its level is affected by inflammation. TS fluctuates due to the diurnal variation of serum iron, and serum iron levels decrease with infection, inflammation, and malignancy and increase with liver disease [2].

Reticulocytes are the youngest erythrocytes released from the bone marrow into the blood and they circulate for 1-2 days before becoming mature erythrocytes. The reticulocytes’ hemoglobin content reflects the amount of iron available for hemoglobin production in the bone marrow. Therefore, reticulocyte hemoglobin content (CHr) has been proposed as an iron status marker [3]. Several studies have indicated that CHr measurement in peripheral blood samples is useful for diagnosis of iron deficiency [4,5,6,7,8]. It has been shown to be an accurate measure of iron status and a reliable iron marker for monitoring iron therapy’s effectiveness [3,9].

In this study, we aimed to evaluate the significance of CHr in the diagnosis of IDA and compare it with other conventional iron parameters.

## MATERIALS AND METHODS

**Patients**

The study was conducted at Eskişehir Osmangazi University, Faculty of Medicine, Department of Hematology. After obtaining the approval of the ethics committee and informed consent, 32 female patients with IDA (serum hemoglobin <120 g/L and serum ferritin <20 ng/mL) and 18 female patients with iron deficiency (serum hemoglobin >120 g/L and serum ferritin <20 ng/mL) were enrolled.

**Sample Collection and Laboratory Methods**

Samples for complete blood count and CHr were collected in K3EDTA tubes and analyzed with an automated hematology analyzer, ADVIA 2120i (Siemens, New York, USA). Serum iron and total iron-binding capacity (TIBC) were measured with a LISA 500 Plus automated chemical analyzer (Hycell Diagnostics, Paris, France). Serum ferritin was measured with a Hitachi E170 automated analyzer (Hitachi, Tokyo, Japan). TS was calculated by dividing serum iron by TIBCx100. Transferrin was measured with a BN II automated chemical analyzer (Siemens, Marburg, Germany).

**Statistical Analysis**

Data were analyzed using IBM SPSS 20. The independent samples t-test was applied for normally distributed variables and results were given as mean ± standard deviation. The Mann-Whitney U test was applied for abnormally distributed variables and results were given as median (quartiles) values. Receiver operating characteristic (ROC) curve analysis was performed to identify the optimal CHr cut-off value for predicting IDA. P<0.05 was accepted as significant.

## RESULTS

There was no statistically significant difference between the 2 groups in terms of age and red blood cell (RBC) count. Hemoglobin, MCV, mean corpuscular hemoglobin (MCH), mean corpuscular hemoglobin concentration (MCHC), serum iron, and TS were significantly lower in female patients with IDA in respect to iron-deficient female patients. Transferrin and TIBC were significantly higher in female patients with IDA compared to female patients with iron deficiency. CHr was 24.95±3.92 pg in the IDA group and 29.93±2.96 pg in the iron deficiency group, and a statistically significant difference was observed between the 2 groups in respect to CHr (Table 1).

CHr showed a significant positive correlation with hemoglobin (r=0.775), MCV (r=0.868), MCH (r=0.883), MCHC (r=0.685), serum iron (r=0.648), and TS (r=0.764) and a significant negative correlation with transferrin (r=-0.599) and TIBC (r=-0.613).

**Figure 1:**
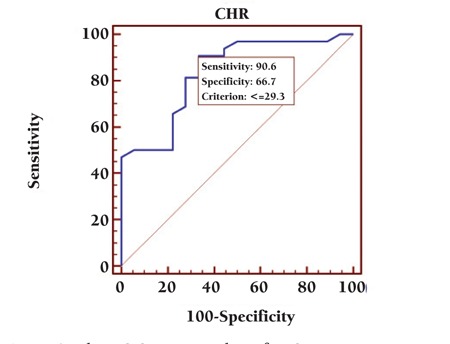
The ROC curve analysis for CHr.

A cut-off value of CHr was determined as 29.3 pg (90.6% sensitivity, 66.7% specificity) by ROC analysis in female patients with IDA anemia (Figure 1). Power analysis of CHr was calculated as 1, which was perfect (NCCS 2007, PASS 2005, and GESS 2006).

## DISCUSSION

Various biochemical parameters are being used for the diagnosis of IDA. However, there might be some difficulties in the assessment of these conventional parameters. For example, ferritin behaves as an acute phase reactant, which limits its diagnostic accuracy greatly. The serum ferritin level is frequently increased independently of iron status by factors such as acute/chronic inflammation, infection, malignancy, liver disease, and alcohol use. Serum iron levels also decrease with infection, inflammation, and malignancy and increase with liver disease. TS is a calculated parameter, and therefore it reflects confounding effects on individual components [2].

Measurement of CHr provides an indirect measure of the functional iron available for new RBC production. In a study performed by Mast et al., it was reported that CHr of <28 pg had an optimal sensitivity (74%) and specificity (73%) for diagnosis of iron deficiency, using Prussian blue staining of the bone marrow aspirate to define iron deficiency. In this study, the area under the curve of CHr exceeded that of ferritin, TS, and MCV, showing that CHr is a useful marker for diagnosis of iron deficiency in adults [4].

Several studies have assessed the value of CHr as an indicator of iron deficiency in dialysis patients. In a recent study by Thomas et al., functional iron deficiency was defined as CHr < 28 pg [10]. Fishbane et al. also reported that CHr of <28 pg predicted iron deficiency more accurately than did serum ferritin and TS in dialysis patients receiving erythropoietin [2]. Mitsuiki et al. reported that a CHr index for iron deficiency with 100% high sensitivity was 32 pg [11]. In another study, a CHr cut-off value of 32 pg was found to be appropriate for the assessment of iron deficiency in hemodialysis patients, in which an ethnic effect on CHr levels was considered to explain the higher cut-off [3]. In our study, a CHr cut-off value of 29 pg with 90.6% sensitivity and 66.7% specificity was determined to predict IDA.

With the present investigation, we have identified the value of CHr in the diagnosis of IDA. CHr showed the strongest correlation with hemoglobin, MCV, MCH, and TS with r>0.7, which was considered meaningful. Therefore, CHr in conjunction with these conventional parameters appears to be useful and reliable in identifying IDA.

In conclusion, besides the current conventional parameters that we use in routine practice to diagnose IDA, there is still a need for more sensitive and powerful parameters. CHr is a useful parameter that can be confidently used in the diagnosis of IDA, and a CHr cut-off value of 29 pg predicts IDA.

**Conflict of Interest Statement**

The authors of this paper have no conflicts of interest, including specific financial interests, relationships, and/or affiliations relevant to the subject matter or materials included.

## Figures and Tables

**Table 1:**
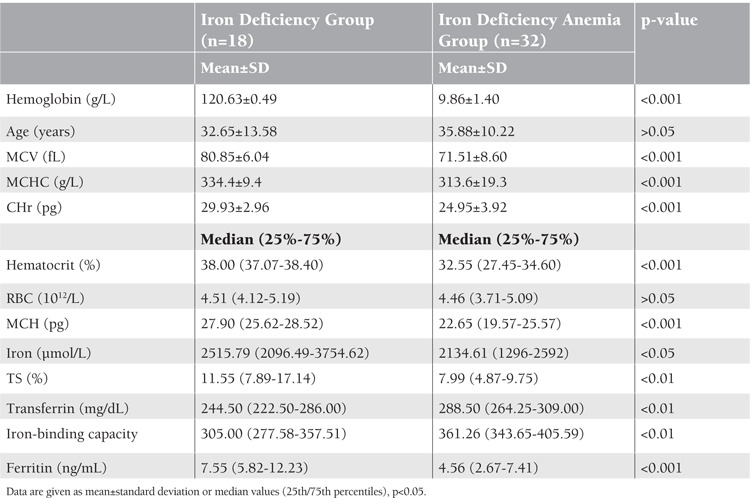
Hematologic and iron parameters in iron deficiency anemia group and iron deficiency group.
